# Satisfaction with healthcare services and related factors among Indonesian migrant workers in Taiwan: a cross-sectional survey study

**DOI:** 10.1186/s12913-025-12722-9

**Published:** 2025-04-23

**Authors:** Ari Setyawan, Hui-Chuan Hsu, Shang-Jyh Chiou, Wen-Chi Wu, Kun-Yang Chuang, Ying-Chih Chuang

**Affiliations:** 1 School of Public Health, College of Public Health, Taipei Medical University, New Taipei City, 235 Taiwan; 2 Department of Health Care Management, College of Health Technology, National Taipei University of Nursing and Health Sciences, Taipei, 112 Taiwan; 3 Department of Health Promotion and Health Education, College of Education, National Taiwan Normal University, Taipei, 106 Taiwan; 4 Department of Public Health, Faculty of Health and Sciences, Universitas Muhammadiyah Prof. Dr. HAMKA, Jakarta, 12160 Indonesia

**Keywords:** Healthcare quality, Quality of care, Patient satisfaction, Migrant worker

## Abstract

**Purpose:**

The purpose of this study was to identify factors associated with Indonesian migrant workers’ satisfaction with healthcare services in Taiwan, and to compare their satisfaction of healthcare between Taiwan and Indonesia.

**Methods:**

This cross-sectional study collected data through self-reported questionnaires from 352 participants. Inclusion criteria of participants were legal Indonesian migrant workers aged 18–55 years who were willing to participate, and only those who had used healthcare services for inpatient, outpatient, or emergency care were included in the analysis (*n* = 241). We used the SERVQUAL model, based on the gap theory of service quality, to assess service quality applied to health care. Analytical methods included descriptive analyses, linear regression, and multinomial logistic regression.

**Results:**

Satisfaction with Taiwan’s health care was high (mean = 4.23 of 5), and almost 70% reported that different dimensions of Taiwan’s health care were better than those of Indonesia. Among the healthcare system factors, longer waiting time was related to lower empathic satisfaction (B=- 0.272), while getting more assistance from volunteers (B = 0.067) and friendliness of staff (B = 0.112) were related to higher tangible satisfaction. When comparing health care in Taiwan to that in Indonesia, longer waiting time was related to higher satisfaction with Indonesia’s healthcare (in tangibility and in responsiveness); expensive health service payment was related to lower satisfaction in Taiwan’ healthcare (OR = 0.432 in tangibility) or higher satisfaction of Indonesia’ healthcare (OR = 5.079 in reliability); and language was related to better satisfaction of Indonesia’s healthcare (OR = 5.277 in tangibility and OR = 10.443 in reliability). Meanwhile, easy explanation was related to lower satisfaction with Indonesia’s healthcare (OR = 0.445 in assurance), and getting volunteer assistance (OR = 0.326 in the tangibility and OR = 0.272 in reliability), and staff friendliness (OR = 0.085 in reliability and OR = 0.216 in empathy) were related to lower satisfaction of Indonesia’s health care. Education and other demographics also related to the comparison of satisfaction. Odds ratios with wide confidence intervals should be explained conservatively.

**Conclusions:**

Individual and healthcare system factors of Indonesian migrant workers influenced the level of their satisfaction with healthcare services in Taiwan in the tangibility and the empathy dimensions. Healthcare providers should consider these factors to improve service quality and migrant worker satisfaction.

**Supplementary Information:**

The online version contains supplementary material available at 10.1186/s12913-025-12722-9.

## Introduction

Patient satisfaction is an evaluation of how satisfied a patient is with the medical care received from their healthcare provider, which is a patient-centered indicator to measure healthcare quality [1]. It is one of the most critical indicators in assessing a healthcare facility’s performance and the overall efficiency of healthcare delivery [2], and it reflects the quality of services provided by healthcare facilities and helps identify potential improvements; delivering services that align with patient expectations is essential to the sustainability and growth of healthcare institutions [3].

Although patient satisfaction was identified as being important for quality of life, viewpoints from vulnerable populations, such as immigrants, are often ignored [4]. Migrant populations generally experience lower satisfaction with healthcare services compared to the general population due to inequities in access to health care [5]. The rating of experiences when using health care for migrants may depend on their cultural backgrounds, access to healthcare, challenges encountered and emotional responses of providers, and delivery in the context of cultural differences and the degree that needs and expectations are met [6, 7]. In this study, we attempted to identify individual- and system-level factors associated with Indonesian migrant workers’ satisfaction with healthcare services in Taiwan.

### The SERVQUAL model

Theories have been used to conceptualize some models, such as Donabedian’s model in structure, process, and outcome [8] and Maxwell’s six dimensions of healthcare quality (effectiveness, acceptability, efficiency, access, equity, and relevance) [9]. The SERVQUAL model [3], based on the gap theory of service quality in business, is one of the most popular methods of assessing service quality applied to health care. According to this model, the quality of healthcare services is assessed by contrasting a patient’s perceptions and expectations from five angles [3]. Tangibles describe how a physical location, tools, staff, and communication tools affect a patient. The environment, also known as services, immediately impacts psychological, sociological, cognitive, and emotional dimensions of health staff and patients. Assurance refers to establishment of credibility and trust through professional services, superior technical expertise, a courteous attitude, and effective communication skills. Responsiveness refers to an assessment of the capacity to resolve issues quickly, effectively address patient complaints, and demonstrate a willingness to assist patients in order to satisfy their needs. Reliability refers to the service provider’s ability to consistently and accurately deliver services, including providing accurate diagnoses, timely treatments, and consistent outcomes. Empathy ensures that patients feel cared for, experience kindness, and always feel welcome at any time and place. The SERVQUAL model has been effectively used over many years to measure the quality of medical services in countries with varying degrees of economic development and showed a good reliability [10], and it is also characterized by examining patient expectations [11]. A rating of better satisfaction with health care using SERVQUAL was highly related to higher overall satisfaction with health care [12, 13].

### Patient satisfaction and related factors

In the recent decade, person-centered care has been emphasized, and patients have grown increasingly knowledgeable and concerned about the quality of treatments. This has encouraged healthcare services to emphasize assessing healthcare quality to fit patients’ expectations [14, 15]. A systematic review categorized factors that influence quality of health care from individual (health beliefs, health literacy, and education), organizational (health policies, practices, and culture), and societal levels (social norms, regulations, and policies) [16]. At the individual (patient) level, a younger age, higher income, higher education, occupation, residence area, coping skills, and preferences may be related to higher perceptions of health care [17–20]. At the organizational (healthcare provider) level are professional competence and knowledge, staff and resource adequacy, nursing practice and management, coworker support, care coordination, physician communication, scheduling and waiting time, and organizational culture [19–22].

### Patient satisfaction of migrant workers

Regarding migrant workers, their patient satisfaction is related to different factors besides the factors above, such as how migrant workers access healthcare services, language limitations, cultural norms, health beliefs, and health-seeking behaviors [23]. Encounters with providers and attitudes of the staff may affect the experience of migrants [7, 24]. In addition, migrants usually have language barriers that may affect their accessibility, doctors’ treatment, and quality of healthcare delivery [25, 26]. A study in China found that the quality of primary health care provided to migrants was not as satisfactory as that offered to local residents, particularly regarding effectiveness, the attitudes of health workers, and waiting time [27].

### Background of Indonesian migrant workers in Taiwan

Indonesian migrant workers are distributed across many countries in Asia, including Taiwan. Taiwan is one of the favored destinations for migrant workers from Southeast Asia, especially Indonesians [28]. In March 1995, the Taiwanese government implemented a national-level health insurance program, the National Health Insurance (NHI) system. The Taiwanese NHI covers not just for Taiwanese but all foreigners with an alien residence certificate (ARC) in Taiwan [29]. Foreigners with an ARC in Taiwan, including students, can apply to enroll in the NHI program through their school [30]. Migrant workers who arrive in Taiwan with a work permit and residence permit can enroll in the NHI as soon as they begin their employment in the country, with no waiting period. In this case, the employer is responsible for their enrolment [31]. Taiwan’s NHI system mandates that once an employee has resided in Taiwan for more than 6 months, employers must offer the same health insurance benefits to international employees as they do to Taiwanese citizens [32]. All legal migrant workers, including Indonesians, are required to have a health screening before applying for an entry visa to Taiwan to prevent the introduction of diseases [33]. Thus, access to healthcare in Taiwan is quite equal among local populations and migrant workers. However, people in Taiwan do not speak Indonesian, and not all medical professionals and migrant workers are fluent in English, which makes communication with Indonesian migrant workers difficult. Second, the culture and the healthcare system in Taiwan are different from those of Indonesia. Third, most hospitals or clinics do not open on weekends. The off days for some migrant workers (for example, in-home domestic workers, care workers, and fishermen) may not be convenient for them to see doctors, and some might not get their wages if they take sick leave.

Although factors affecting patient satisfaction with health care have been documented, some research gaps exist. First, the perception of healthcare quality and satisfaction of migrant workers in the recipient countries are little explored in the literature. Most of the past studies only focused on access of health care services. Second, satisfaction with health services is usually based on the gap between expectation and perception. Little research has compared the experiences of health care services between the mother country and the migrant workers’ country. Third, the SERVQUAL scale, although has been applied widely, is not used among the migrant worker population yet. If the SERVQUAL scale is applied and the comparison can be done, the results can be applied to improve the provision of healthcare for migrant workers. Therefore, the purpose of the current study was to identify factors associated with Indonesian migrant workers’ satisfaction with healthcare services in Taiwan, and to compare their satisfaction of healthcare between Taiwan and Indonesia. We applied the SERVQUAL model to measure the quality of healthcare services for Indonesian migrant workers. The findings are expected to provide policy implications to improve the quality of healthcare services for migrant workers.

## Methods

### Data and sample

The study had a cross-sectional study design. Participants were invited by purposive sampling. In Taiwan, most Indonesian migrant workers are Muslim, and they attend Muslim religious activities regularly. Thus, Indonesian migrant workers were reached through Muslim religious communities during their activities in five cities in Taiwan. Inclusion criteria were as follows: a legal Indonesian migrant; a full-time worker; aged 18–55 years; occupation in manufacturing, as a crewman, or in the human social service field; at least once experience in using health care in Taiwan and Indonesia; and willing and able to fill out the questionnaire. There were no exclusion criteria. Only those who had used inpatient, outpatient, or emergency healthcare services in the last year were included for analysis. Data were collected through a self-written questionnaire explaining the research by the first author in the religious activities from August to October 2023. The questionnaire draft was reviewed by experts and revised to ensure content validity. The questionnaire was designed in English, translated into Bahasa Indonesian, and then back-translated into English to ensure accuracy. A pre-test was first conducted; then the questionnaire was revised based on results of the validation and pre-test. We used G-Power 3.1.9.2 software [34] to estimate the necessary sample size. The test family = F test, statistical test = linear multiple regression- omnibus (deviation or R^2^ from zero), fixed model, type of power analysis: a priori (compute required sample size– given α, power, and effect size). We used the F test of multiple linear regression– Omnibus method (deviation of R^2^ from zero, fixed model) to estimate the minimum sample size: we set the power (1- β) level = 0.8, effect size ƒ^2^= 0.2, α level = 0.05, number of predictors = 22, and then the minimum sample size would be 127. In total, 352 participants completed the questionnaire. Only those who has used health care in the past year were analyzed in this study (*n* = 241).

### Measures

#### Individual characteristics

Individual characteristics included age, gender (male or female), marital status (with a spouse or no spouse), educational level (junior high school and lower or senior high school and higher), and occupation (manufacturing, crewman, or human social services). Enabling factors included income level (<$635 US dollars/month or ≥ $635 US dollars/month, as $635 dollars/month was the minimum salary requirement for workers in Taiwan), insurance status (indicating coverage by Taiwan’s NHI: yes or no/I don’t know), time spent in Taiwan (≤ 2 or > 2 years), and if they need a translator when using health care (no or yes). The health status was measured by the 12-item Short-Form Health Survey (SF- 12), a self-reported outcome measure assessing the impact of health on an individual’s life. The scoring yields two summary measures: the Physical Component Summary (PCS) and the Mental Component Summary (MCS) [35]. A score of ≤ 50 on the PCS- 12 was recommended as a cutoff to determine poor physical health, while a score of ≤ 42 on the MCS- 12 may indicate poor mental health [34].

#### Organizational factors of healthcare providers

Factors of healthcare providers included accessibility (waiting time to make an appointment, counseling times, waiting time for the doctor, transportation times, and payments for use of health services) and friendliness for migrants (explanation by providers, language communication of providers, availability of translation services, assistance by volunteers, and friendly attitudes of healthcare staff). Waiting time (to make an appointment) was assessed by asking how long was the duration between making an appointment and seeing the doctor or being admitted. Counseling time was measured by asking the number of minutes or hours spent by patients with healthcare staff. Waiting time for the doctor was assessed by asking how long a patient needed to wait for the doctor until meeting with them coded as 1 (very short) to 5 (very long). Transportation time was assessed by asking about the time it took to access and obtain healthcare services coded as 1 (very short) to 5 (very long). Health service payment was assessed by asking how much was needed to pay for a single service coded as 1 (very cheap) to 5 (very expensive). Friendliness toward migrants was assessed by evaluating the following factors [35] (with each item scored from 1 to 5): whether the providers gave an easy explanation (never to all of the time), the healthcare staff’s communication ability (poor to good), the availability of translation services during communication with healthcare staff (never to all of the time), the availability of special assistance by volunteers for migrants (never to all of the time), and whether the attitudes of the staff and healthcare workers were friendly (poor to good).

#### Satisfaction with health care

Satisfaction with healthcare quality was evaluated using the SERVQUAL scale [3], and questions were modified based on findings of the pre-test. After the pre-test, only 21 items of the SERVQUAL scale were applied. The five dimensions and items were as follows. (1) Tangibility measures were about how well the physical tools, environment, and staff affected the patient’s experience. (2) Reliability assessed the reliability of healthcare workers, including doing the promised service on time, showing interest in solving problems, doing everything right the first time, fulfilling promised services at the promised time, and keeping accurate records and documents. Two items in the original SERVQUAL scale about the promise of care were similar according to participants in the pre-test, and thus the two items were combined into one question. (3) The responsiveness dimension evaluates how well healthcare staff responded to patient requests, including announcing exact service times, providing fast and prompt service, being willing to help, and being available when needed. (4) Assurance measures attitudes of healthcare workers, including trustful behavior, a sense of security and comfort, politeness and humility, and knowledge to answer questions. (5) Empathy assesses the attention given by healthcare workers to patients, including individual attention, convenient hours, understanding specific needs, providing services according to the patient’s interests, and paying equal attention to all patients regardless of their social status. The items were coded from 1 to 5 (1 = very disagree; 2 = disagree; 3 = neutral; 4 = agree 5 = very agree). Items were summed-up for a total score defined as total satisfaction with health care. The internal consistency of the total SERVQUAL scale was high: Cronbach’s alpha of the total scale was 0.91, and Cronbach’s alpha for each dimension ranged 0.74–0.87 (please the Appendix Table S1).

We also asked migrant worker participants to compare their satisfaction with using health care in terms of the environment (tangibility), handling patients reliably (reliability), responsiveness, trustworthiness (assurance), and attention (empathy) between Taiwan and Indonesia. Satisfaction with healthcare was defined as the gap between perceptions and expectations [3]. We assume every migrant worker has at least once experienced using healthcare in Indonesia. Thus, satisfaction with Indonesia health care was used as the expectation, and healthcare in Taiwan was defined as the expectation. The response was coded into three groups: Taiwan is better, Indonesia is better, or they are similar or not sure.

### Analysis

Data were analyzed using the Statistical Package for Social Sciences (SPSS) program software vers. 29.0 (IBM, SPSS, Armonk, NY, USA). The analytical methods included descriptive analysis, multiple linear regression, and multinomial logistic regression. Multiple regression was used to examine individual and healthcare system factors related to overall satisfaction and satisfaction by dimensions of the SERVQUAL scale, and multinomial logistic regression was used to compare of satisfaction between Taiwan and Indonesia and explore its factors.

### Ethical considerations

The protocol for this study was approved by the ethics committee of the TMU Institutional Review Board (no. N202306075). We obtained informed consent from each respondent before administering the survey. The first author explained and obtained the research to get the informed consent of the participants. All the questionnaires were self-filled in Indonesian and anonymous.

## Results

Table 1 shows results of the descriptive analysis of individual and healthcare system characteristics among Indonesian migrant workers in Taiwan. The gender distribution of the sample was 54.8% males and 45.2% females. Their primary occupation was manufacturing (45.2%), followed by human social services (39.8%) and a smaller portion in the crewman sector (14.9%). Almost all respondents were covered by Taiwan’s NHI (96.7%). As to organizational factors, most of the participants reported the transportation time to healthcare facilities was short (93.4%); 27.8% of respondents found explanations provided by healthcare professionals easy to understand, and 54.8% rated the language proficiency of healthcare providers as good. However, only 83.0% reported that translator services were provided all of the time. Only 23.2% of respondents had the experience of being assisted by volunteers, and 84.2% expressed good friendliness of staff.

**Table 1:** Characteristics of the participants among Indonesian migrant workers in Taiwan

Variables	Frequency (*n*)	Percent (%) or Mean (SD)
**Individual factors**		
Sex		
Male	132	54.8
Female	109	45.2
Age group		
< 30 years	109	45.2
≥ 30 years	131	54.4
Marital status		
Having spouse	138	57.3
No having spouse	101	41.9
Occupation		
Manufacturing	109	45.2
Crewman	36	14.9
Human social service	96	39.8
Income level		
< $635 US dollars/month	42	17.4
≥ $635 US dollars/month	198	82.2
Educational Level		
Junior high school or lower	92	38.2
Senior high school or higher	141	58.5
Taiwan NHI status		
Yes	233	96.7
No/I don’t know	8	3.3
Time spent in Taiwan		
≤ 2 years	69	28.6
> 2 years	165	68.5
Translator needed		
No	119	49.4
Yes	122	50.6
Physical health		
Poor	59	24.5
Good	181	75.1
Mental health		
Poor	120	49.8
Good	120	49.8
**Healthcare system factors**		
Waiting time to make an appointment		
Less than one day	225	93.4
More than one day	16	6.6
Counseling time		
Less than one hour	217	90.0
More than one hour	24	10.0
Need to wait the doctor (1 ~ 5)	241	2.13 (0.607)
Time of transportation (1 ~ 5)	241	2.14 (0.675)
Payment of using health service (1 ~ 5)	241	2.05 (0.625)
Easy-to-understand explanations (1 ~ 5)	241	3.54 (1.207)
Language (1 ~ 5)	241	3.65 (0.703)
Translator provided (1 ~ 5)	241	2.17 (1.321)
Get assistance by volunteers (1 ~ 5)	241	2.43 (1.293)
Friendliness of staff (1 ~ 5)	241	4.05 (0.526)

*N* = 241, missing cases were listwise deleted in the percentages

**Fig. 1:**
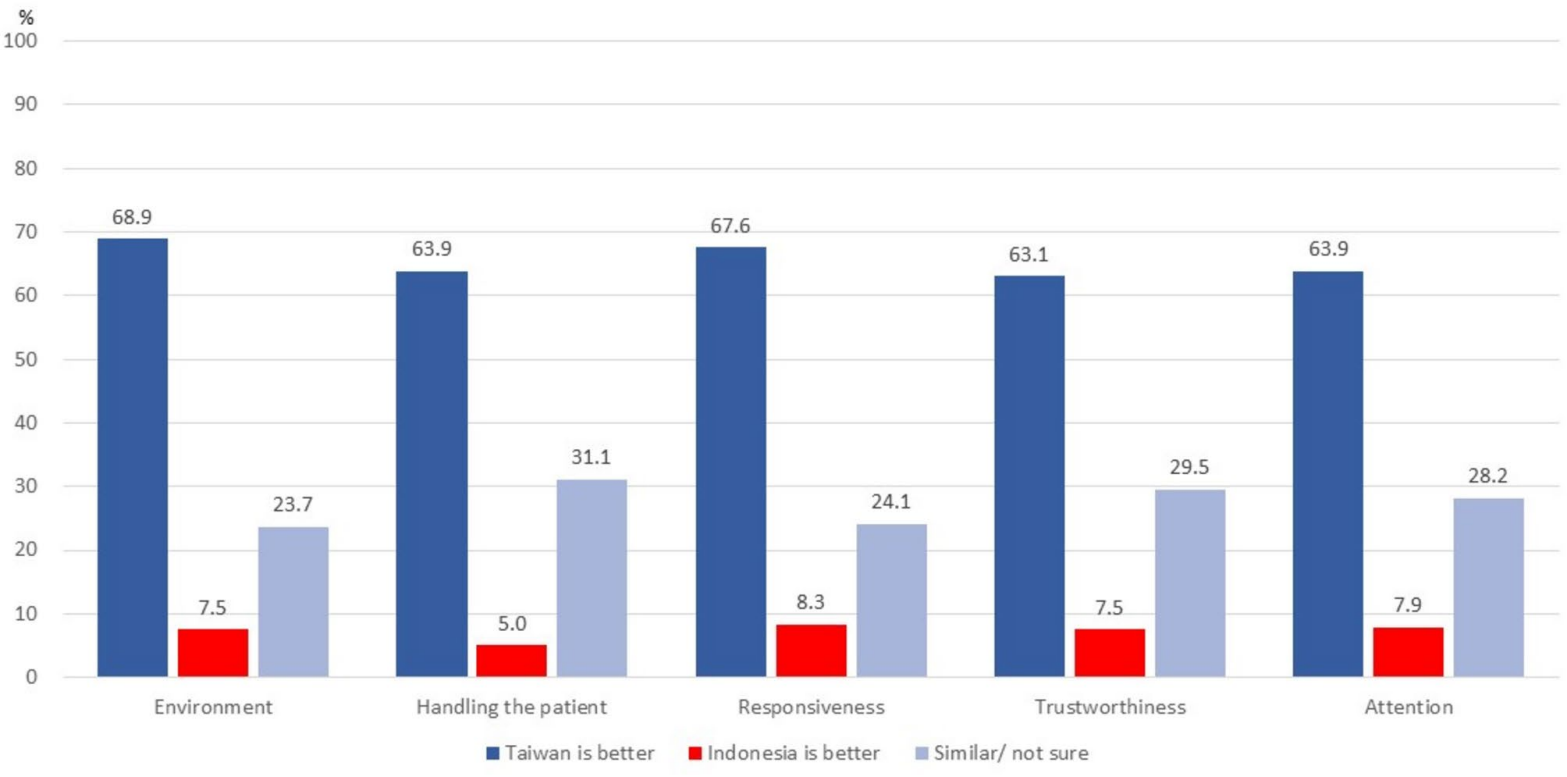
Percentage of health care satisfaction comparison between Taiwan and Indonesia

Table 2 shows the distribution of satisfaction using the SERVQUAL scale and gaps in healthcare utilization satisfaction among Indonesian migrant workers in Taiwan. The average scores of satisfaction for all of the items and by dimension were very close, ranging 4.00–4.39. The overall mean satisfaction score was 4.23. Comparing healthcare service quality gaps between Taiwan and Indonesia, the majority (from 63.9 to 68.9%) found that Taiwan’s healthcare service was better than Indonesia’s across aspects of environment, handling of patients, responsiveness, trustworthiness, and attention (please see Fig. 1).

**Table 2:** Satisfaction with health care utilization and gaps among Indonesian migrant workers in Taiwan

Variables	Percent (%) or Mean (SD)
**Overall satisfaction**	4.23 (0.314)
**Tangibility**	4.27 (0.422)
Equipment (1 ~ 5)	4.32 (0.503)
Physical environment (1 ~ 5)	4.16 (0.683)
Dressed and neat of Uniform (1 ~ 5)	4.33 (0.537)
Appealing (1 ~ 5)	4.31 (0.497)
**Reliability**	4.22 (0.399)
Willing to solve (1 ~ 5)	4.23 (0.564)
Do everything right (1 ~ 5)	4.18 (0.444)
Fulfill their promised (1 ~ 5)	4.20 (0.467)
Keep accurate records (1 ~ 5)	4.28 (0.520)
**Responsiveness**	4.20 (0.437)
Announce the exact time (1 ~ 5)	4.23 (0.487)
Fast and promptly (1 ~ 5)	4.19 (0.537)
Willing to help (1 ~ 5)	4.21 (0.481)
Respond to patients (1 ~ 5)	4.17 (0.563)
**Assurance**	4.25 (0.409)
x2003;Trustworthy (1 ~ 5)	4.28 (0.449)
Patients feel safe (1 ~ 5)	4.25 (0.461)
Polite and humble (1 ~ 5)	4.26 (0.494)
Knowledgeable enough (1 ~ 5)	4.22 (0.520)
**Empathy**	4.21 (0.381)
Shown individual attention (1 ~ 5)	4.22 (0.412)
The hour is convenient (1 ~ 5)	4.00 (0.636)
Understand the patients (1 ~ 5)	4.17 (0.460)
Provide services of need (1 ~ 5)	4.26 (0.438)
Irrespective of the social status (1 ~ 5)	4.39 (0.561)

*N* = 241, missing cases were listwise deleted in the percentages

Table 3 shows results of the multiple linear regression analysis of various factors associated with overall satisfaction and satisfaction by dimensions of the SERVQUAL scale among Indonesian migrant workers in Taiwan. In terms of healthcare system characteristics. Females had lower satisfaction with the responsiveness dimension (B=− 0.251, *p* < 0.01). Those who were aged ≥ 30 years expressed higher overall satisfaction (B = 0.136, *p* < 0.05) in the tangible dimension (B = 0.172, *p* < 0.01) and the responsiveness dimension (B = 0.188). Those without a spouse had higher satisfaction in the reliability dimension (B = 0.156, *p* < 0.05). Those with a higher income (≥ $635 US dollars/month) had lower satisfaction in the responsiveness dimension (B=− 0.217, *p* < 0.05). A longer waiting time to get an appointment was related to lower satisfaction in the empathy dimension (B=− 0.272, *p* < 0.05). Having been provided more volunteer assistance was related to higher satisfaction in the tangible dimension (B = 0.067, *p* < 0.01). Better staff friendliness was related to higher satisfaction in the tangible dimension (B = 0.112, *p* < 0.05).

**Table 3:** Satisfaction with healthcare in Taiwan with individual and health care system factors by linear regression

Variables	Overall Satisfaction	Tangible Dimension	Reliability Dimension	Responsiveness Dimension	Assurance Dimension	Empathy Dimension
	B (SE)	B (SE)	B (SE)	B (SE)	B (SE)	B (SE)
**Individual factors**						
Sex						
Male	*Reference*	*Reference*	*Reference*	*Reference*	*Reference*	*Reference*
Female	− 0.091 (0.069)	− 0.004 (0.091)	− 0.090 (0.090)	− 0.251 (0.091)^**^	− 0.077 (0.091)	− 0.047 (0.084)
Age						
< 30 years	*Reference*	*Reference*	*Reference*	*Reference*	*Reference*	*Reference*
≥ 30 years	0.136 (0.052)^*^	0.172 (0.069)^*^	0.133 (0.068)	0.188 (0.069)^**^	0.122 (0.068)	0.078 (0.063)
Marital status						
Having spouse	*Reference*	*Reference*	*Reference*	*Reference*	*Reference*	*Reference*
No having spouse	0.080 (0.050)	0.068 (0.067)	0.156 (0.066)^*^	0.113 (0.066)	0.080 (0.066)	0.002 (0.061)
Occupation						
Manufacturing	*Reference*	*Reference*	*Reference*	*Reference*	*Reference*	*Reference*
Crewman	− 0.055 (0.071)	− 0.071 (0.094)	− 0.048 (0.093)	0.095 (0.094)	− 0.127 (0.094)	− 0.109 (0.087)
Human social service	0.003 (0.072)	0.029 (0.096)	0.014 (0.095)	0.109 (0.096)	− 0.068 (0.095)	− 0.055 (0.088)
Income level						
< $635 US dollars/month	*Reference*	*Reference*	*Reference*	*Reference*	*Reference*	*Reference*
≥ $635 US dollars/month	− 0.097 (0.064)	0.029 (0.085)	− 0.060 (0.084)	− 0.217 (0.085)^*^	− 0.111 (0.084)	− 0.118 (0.078)
Educational Level						
Junior high school or lower	*Reference*	*Reference*	*Reference*	*Reference*	*Reference*	*Reference*
Senior high school or higher	0.047 (0.047)	0.057 (0.062)	0.022 (0.061)	0.029 (0.062)	0.050 (0.062)	0.070 (0.057)
Taiwan NHI status						
Yes	*Reference*	*Reference*	*Reference*	*Reference*	*Reference*	*Reference*
No/I don’t know	0.071 (0.118)	0.225 (0.156)	0.146 (0.155)	0.209 (0.156)	− 0.040 (0.155)	− 0.132 (0.144)
Time spent in Taiwan						
≤ 2 years	*Reference*	*Reference*	*Reference*	*Reference*	*Reference*	*Reference*
> 2 years	− 0.035 (0.050)	− 0.113 (0.066)	− 0.018 (0.065)	− 0.009 (0.066)	− 0.051 (0.065)	0.006 (0.060)
Translator needed						
No	*Reference*	*Reference*	*Reference*	*Reference*	*Reference*	*Reference*
Yes	− 0.016 (0.046)	0.032 (0.061)	− 0.026 (0.060)	− 0.056 (0.061)	− 0.021 (0.061)	− 0.011 (0.056)
Physical health						
Poor	*Reference*	*Reference*	*Reference*	*Reference*	*Reference*	*Reference*
Good	0.035 (0.051)	− 0.041 (0.068)	0.042 (0.067)	0.103 (0.068)	0.060 (0.068)	0.018 (0.063)
Mental health						
Poor	*Reference*	*Reference*	*Reference*	*Reference*	*Reference*	*Reference*
Good	0.059 (0.046)	− 0.016 (0.061)	0.051 (0.060)	0.053 (0.061)	0.115 (0.061)	0.084 (0.056)
**Healthcare system factors**						
Waiting time to make an appointment						
Less than one day	*Reference*	*Reference*	*Reference*	*Reference*	*Reference*	*Reference*
More than one day	− 0.129 (0.095)	− 0.081 (0.126)	− 0.094 (0.125)	− 0.012 (0.126)	− 0.152 (0.125)	− 0.272 (0.116)^*^
Counselling time						
Less than one hour	*Reference*	*Reference*	*Reference*	*Reference*	*Reference*	*Reference*
More than one hour	0.030 (0.083)	0.141 (0.110)	0.065 (0.109)	− 0.165 (0.110)	− 0.027 (0.109)	0.115 (0.101)
Need to wait the doctor (long)	− 0.043 (0.042)	− 0.031 (0.056)	− 0.032 (0.055)	− 0.027 (0.055)	− 0.040 (0.055)	− 0.074 (0.051)
Time of transportation (long)	− 0.005 (0.036)	− 0.026 (0.047)	− 0.002 (0.047)	− 0.037 (0.047)	− 0.003 (0.047)	0.032 (0.043)
Health service payment (expensive)	− 0.033 (0.036)	− 0.093 (0.048)	− 0.017 (0.048)	− 0.089 (0.048)	− 0.018 (0.048)	0.036 (0.044)
Easy explanations (often)	0.019 (0.020)	0.037 (0.027)	0.028 (0.027)	− 0.001 (0.027)	8.570E (0.027)	0.028 (0.025)
Language (good)	0.044 (0.033)	0.023 (0.044)	0.035 (0.043)	0.063 (0.044)	0.030 (0.044)	0.063 (0.040)
Translator provided (often)	− 0.030 (0.018)	− 0.036 (0.024)	− 0.025 (0.024)	− 0.038 (0.024)	− 0.024 (0.024)	− 0.029 (0.022)
Assist by volunteers (often)	0.028 (0.018)	0.067 (0.025)^**^	0.020 (0.024)	0.028 (0.024)	0.010 (0.024)	0.019 (0.023)
Friendliness of staff (good)	0.046 (0.041)	0.112 (0.055)^*^	0.090 (0.054)	0.041 (0.055)	− 0.002 (0.055)	0.001 (0.050)

*N* = 241. Analysis by linear regression

^*^p < 0.05

^**^p < 0.01

Table 4 shows results of the comparison of satisfaction of health care between Taiwan and Indonesia by the multinomial logistic regression; the reference group was “similar”. Regarding individual factors, females were less likely to rate Indonesia’s tangibility as better (OR = 0.021, *p* < 0.05). Those aged ≥ 30 years were more likely to rate Taiwan’s system in tangibility as better (OR = 2.900, *p* < 0.05) and less likely to rate Indonesia’s system more trustworthy of assurance (OR = 0.178, *p* < 0.05). Individuals without a spouse were more likely to rate Taiwan’s tangibility (OR = 4.071, *p* < 0.05) and reliability (OR = 2.356, *p* < 0.05) as better. Compared to workers in manufacturing, crewmen were more likely to rate Indonesia’s tangibility (OR = 8.820, *p* < 0.05) and Indonesia’s empathy (OR = 14.845, *p* < 0.01) as better, and those in human social services favored Indonesia’s tangibility (OR = 20.558, *p* < 0.05). Higher-income workers were more likely to rate Taiwan’s empathy as better (OR = 3.021, *p* < 0.05). Those with higher education levels were generally less satisfied with Indonesia’s healthcare across multiple dimensions, including tangibility (OR = 0.068, *p* < 0.05), reliability (OR = 0.028, *p* < 0.01), assurance (OR = 0.140, *p* < 0.05), and empathy (OR = 0.182, *p* < 0.05), while being generally less satisfied with Taiwan’s health care across multiple dimensions, including reliability (OR = 0.396, *p* < 0.05), responsiveness (OR = 0.291, *p* < 0.01), assurance (OR = 0.368, *p* < 0.01) and empathy (OR = 0.308, *p* < 0.01). Workers who needed a translator were less likely to rate Taiwan’s tangibility as better (OR = 0.247, *p* < 0.01).

**Table 4:** Comparison of satisfaction with healthcare by dimension between Taiwan and Indonesia and related factors by multinomial logistic regression (crude odds ratio)

Variables	Tangibility		Reliability		Responsiveness		Assurance		Empathy	
	Taiwan is better	Indonesia is better	Taiwan is better	Indonesia is better	Taiwan is better	Indonesia is better	Taiwan is better	Indonesia is better	Taiwan is better	Indonesia is better
**Individual factors**										
Sex										
Male	*Reference*	*Reference*	*Reference*	*Reference*	*Reference*	*Reference*	*Reference*	*Reference*	*Reference*	*Reference*
Female	1.553 (0.415–5.803)	0.021 (0.001–0.587)^*^	1.036 (0.379–2.833)	0.022 (0.00–3.822)	0.884 (0.305–2.556)	0.196 (0.018–2.116)	0.625 (0.228–1.713)	0.240 (0.022–2.624)	1.143 (0.414–3.156)	0.125 (0.007–2.387)
Age										
< 30 years	*Reference*	*Reference*	*Reference*	*Reference*	*Reference*	*Reference*	*Reference*	*Reference*	*Reference*	*Reference*
≥ 30 years	2.900 (1.160–7.252)^*^	0.452 (0.072–2.817)	1.362 (0.631–2.937)	0.128 (0.011–1.463)	1.381 (0.605–3.15)	0.364 (0.087–1.523)	1.111 (0.517–2.385)	0.178 (0.032–0.998)^*^	1.048 (0.485–2.264)	0.582 (0.115–2.951)
Marital status										
Having spouse	*Reference*	*Reference*	*Reference*	*Reference*	*Reference*	*Reference*	*Reference*	*Reference*	*Reference*	*Reference*
No having spouse	4.071 (1.536–10.792)^*^	4.766 (0.824–27.554)	2.356 (1.070–5.183)^*^	6.289 (0.668–59.219)	1.135 (0.495–2.602)	0.803 (0.198–3.252)	1.379 (0.643–2.958)	1.563 (0.324–7.537)	0.909 (0.421–1.962)	1.416 (0.265–7.555)
Occupation										
Manufacturing	*Reference*	*Reference*	*Reference*	*Reference*	*Reference*	*Reference*	*Reference*	*Reference*	*Reference*	*Reference*
Crewman	2.406 (0.631–9.174)	8.820 (1.075–72.387)^*^	0.857 (0.291–2.519)	9.801 (0.86–111.652)	0.679 (0.211–2.181)	3.492 (0.581–20.989)	0.503 (0.172–1.468)	4.044 (0.611–26.786)	0.676 (0.208–2.195)	14.845 (1.962–112.314)^**^
Human social service	2.111 (0.518–8.610)	20.558 (1.00–419.353)^*^	1.267 (0.435–3.688)	3.454 (0.045–267.58)	1.746 (0.569–5.36)	1.026 (0.083–12.724)	1.755 (0.610–5.049)	1.695 (0.137–20.965)	1.251 (0.435–3.599)	3.556 (0.199–63.594)
Income level										
< $635 US dollars/month	*Reference*	*Reference*	*Reference*	*Reference*	*Reference*	*Reference*	*Reference*	*Reference*	*Reference*	*Reference*
≥ $635 US dollars/month	1.324 (0.422–4.154)	0.973 (0.098–9.644)	1.961 (0.777–4.944)	4.973 (0.132–186.7)	2.028 (0.74–5.558)	0.982 (0.154–6.258)	1.868 (0.726–4.808)	1.159 (0.159–8.438)	3.021 (1.147–7.953)^*^	3.319 (0.371–29.726)
Educational Level										
Junior high school or lower	*Reference*	*Reference*	*Reference*	*Reference*	*Reference*	*Reference*	*Reference*	*Reference*	*Reference*	*Reference*
Senior high school or higher	0.900 (0.385–2.108)	0.068 (0.010–0.469)^*^	0.396 (0.189–0.83)^*^	0.028 (0.002–0.39)^**^	0.291 (0.125–0.679)^**^	0.251 (0.061–1.033)	0.368 (0.175–0.776)^**^	0.140 (0.029–0.674)^*^	0.308 (0.141–0.671)^**^	0.182 (0.037–0.893)^*^
Taiwan NHI status										
Yes	*Reference*	*Reference*	*Reference*	*Reference*	*Reference*	*Reference*	*Reference*	*Reference*	*Reference*	*Reference*
No/I don’t know	3.830 (0.280–52.376)	1.679 (0.059–48.033)	0.48 (0.096–2.403)	1.390 (1.390–1.390)	0.393 (0.069–2.234)	0.764 (0.045–13.061)	0.356 (0.070–1.823)	2.580 (2.58–2.58)	1.021 (0.160–6.520)	3.180 (0.121–83.404)
Time spent in Taiwan										
≤ 2 years	*Reference*	*Reference*	*Reference*	*Reference*	*Reference*	*Reference*	*Reference*	*Reference*	*Reference*	*Reference*
> 2 years	1.026 (0.438–2.406)	0.901 (0.175–4.643)	1.295 (0.627–2.675)	0.537 (0.076–3.813)	1.311 (0.614–2.798)	1.838 (0.479–7.057)	1.504 (0.736–3.070)	2.347 (0.500–11.016)	1.330 (0.647–2.735)	1.931 (0.412–9.050)
Translator needed										
No	*Reference*	*Reference*	*Reference*	*Reference*	*Reference*	*Reference*	*Reference*	*Reference*	*Reference*	*Reference*
Yes	0.247 (0.104–0.589)^**^	0.667 (0.148–3.001)	0.678 (0.341–1.351)	3.402 (0.441–26.229)	0.792 (0.373–1.681)	1.222 (0.331–4.505)	0.862 (0.428–1.736)	1.303 (0.330–5.140)	0.938 (0.461–1.908)	3.535 (0.747–16.735)
Physical health										
Poor	*Reference*	*Reference*	*Reference*	*Reference*	*Reference*	*Reference*	*Reference*	*Reference*	*Reference*	*Reference*
Good	1.557 (0.635–3.819)	0.873 (0.199–3.833)	1.635 (0.766–3.49)	0.333 (0.041–2.671)	2.14 (0.937–4.891)	1.248 (0.313–4.978)	1.189 (0.545–2.595)	1.065 (0.231–4.905)	1.102 (0.494–2.457)	0.729 (0.153–3.470)
Mental health										
Poor	*Reference*	*Reference*	*Reference*	*Reference*	*Reference*	*Reference*	*Reference*	*Reference*	*Reference*	*Reference*
Good	0.622 (0.269–1.436)	1.027 (0.210–5.014)	1.185 (0.592–2.372)	2.816 (0.368–21.567)	0.866 (0.407–1.845)	0.503 (0.126–2.003)	1.150 (0.577–2.290)	0.842 (0.194–3.666)	1.110 (0.547–2.251)	0.362 (0.072–1.813)
**Healthcare system factors**										
Waiting time										
Less than one day	*Reference*	*Reference*	*Reference*	*Reference*	*Reference*	*Reference*	*Reference*	*Reference*	*Reference*	*Reference*
More than one day	1.734 (0.255–11.782)	28.767 (1.110–745.472)^*^	1.027 (0.243–4.337)	1.546 (0.006–407.134)	0.571 (0.124–2.628)	12.428 (1.084–142.53)^*^	0.350 (0.085–1.452)	4.292 (0.231–79.591)	0.764 (0.185–3.161)	2.117 (0.087–51.215)
Counselling time										
Less than one hour	*Reference*	*Reference*	*Reference*	*Reference*	*Reference*	*Reference*	*Reference*	*Reference*	*Reference*	*Reference*
More than one hour	1.121 (0.246–5.105)	0.734 (0.043–12.445)	1.44 (0.4–5.188)	0.601 (0.02–17.787)	1.052 (0.285–3.88)	0.117 (0.006–2.242)	1.627 (0.450–5.882)	0.127 (0.003–5.637)	0.841 (0.238–2.976)	0.289 (0.021–4.029)
Need to wait the doctor (long)	0.905 (0.449–1.824)	0.544 (0.128–2.308)	0.92 (0.496–1.707)	1.275 (0.112–14.499)	1.187 (0.572–2.463)	1.378 (0.393–4.831)	1.246 (0.616–2.520)	1.380 (0.405–4.701)	1.450 (0.705–2.982)	1.817 (0.516–6.396)
Time of transportation (long)	1.499 (0.749–2.999)	0.969 (0.261–3.599)	1.139 (0.664–1.954)	0.063 (0.005–0.747)*	1.375 (0.721–2.625)	0.432 (0.121–1.548)	1.309 (0.736–2.329)	0.931 (0.329–2.632)	1.459 (0.790–2.693)	0.463 (0.129–1.660)
Health service payment (expensive)	0.432 (0.217–0.861)^*^	2.380 (0.827–6.850)	0.588 (0.329–1.05)	5.079 (1.192–21.642)^*^	0.604 (0.319–1.144)	1.386 (0.617–3.114)	0.658 (0.357–1.212)	2.021 (0.875–4.667)	0.748 (0.403–1.388)	2.341 (0.945–5.801)
Easy explanations (often)	1.086 (0.760–1.553)	0.681 (0.336–1.378)	1.023 (0.758–1.382)	0.822 (0.288–2.348)	1.150 (0.828–1.598)	0.703 (0.383–1.29)	1.106 (0.816–1.500)	0.445 (0.217–0.911)^*^	1.235 (0.907–1.683)	0.604 (0.299–1.220)
Language (good)	1.152 (0.655–2.026)	5.277 (1.459–19.084)^*^	1.291 (0.799–2.086)	10.443 (1.7–64.165)^*^	1.26 (0.748–2.121)	2.02 (0.706–5.777)	1.445 (0.883–2.366)	2.028 (0.698–5.897)	1.288 (0.784–2.114)	1.432 (0.421–4.874)
Translator provided (often)	0.949 (0.685–1.316)	1.576 (0.881–2.821)	1.007 (0.764–1.328)	1.384 (0.673–2.844)	1.021 (0.752–1.385)	1.612 (0.974–2.669)	0.972 (0.738–1.280)	0.966 (0.531–1.755)	0.956 (0.723–1.264)	0.890 (0.472–1.678)
Assist by volunteers (often)	0.931 (0.674–1.284)	0.326 (0.153–0.694)^**^	0.857 (0.649–1.132)	0.272 (0.099–0.749)^*^	0.810 (0.597–1.099)	0.656 (0.378–1.137)	0.812 (0.615–1.073)	0.651 (0.341–1.242)	0.878 (0.657–1.174)	1.059 (0.571–1.964)
Friendliness of staff (good)	0.938 (0.387–2.277)	0.754 (0.155–3.657)	0.743 (0.363–1.521)	0.085 (0.008–0.877)^*^	1.239 (0.593–2.587)	0.374 (0.087–1.598)	1.173 (0.608–2.265)	0.711 (0.169–2.998)	0.632 (0.296–1.350)	0.216 (0.047–1.000)^*^

*N* = 241. Analysis by multinomial logistic regression

Reference group of the outcome: satisfaction (similar), ^*^p < 0.05, ^**^p < 0.01

Regarding healthcare system factors, longer waiting time was associated with higher satisfaction for Indonesia’s tangibility (OR = 28.767, *p* < 0.05) and responsiveness (OR = 12.428, *p* < 0.05). More-expensive health service payments were related to lower satisfaction with Taiwan’s tangibility (OR = 0.432, *p* < 0.05) and higher satisfaction with Indonesia’s reliability (OR = 5.079, *p* < 0.05). Getting easy explanations more frequently was related to lower satisfaction with Indonesia’s assurance (OR = 0.445, *p* < 0.05). Accessing better language services were associated with higher satisfaction for Indonesia’s tangibility (OR = 5.277, *p* < 0.05) and reliability (OR = 10.443, *p* < 0.05). More-frequent assistance by volunteers was related to lower satisfaction with Indonesia’s tangibility (OR = 0.326. *p* < 0.01) and reliability (OR = 0.272, *p* < 0.05). Those who were often assisted by volunteers were less likely to rate Indonesia’s tangibility (OR = 0.326, *p* < 0.01) and reliability (OR = 0.272, *p* < 0.05) as better. More friendliness by staff was associated with lower satisfaction with Indonesia’s reliability (OR = 0.085, *p* < 0.05) and empathy (OR = 0.216, *p* < 0.05). These findings highlight the complex interplay of factors influencing migrant workers’ comparative healthcare satisfaction between their host and home countries.

## Discussion

This study examined individual and healthcare system factors associated with overall satisfaction and satisfaction by dimensions of the SERVQUAL scale among Indonesian migrant workers in Taiwan. In general, participants had high satisfaction for Taiwan’s health care. Longer waiting time were related to lower satisfaction with empathy, while volunteer assistance and friendliness were related to higher satisfaction with tangibles. When comparing satisfaction with healthcare between Taiwan and Indonesia, almost 70% of participants reported Taiwan’s health care was better. When comparing health care in Taiwan and Indonesia, waiting time, more-expensive payments, and provider’s language abilities were related to higher satisfaction with Indonesia’ health care (or lower satisfaction with Taiwan’s) in environment and handling patients; easy explanations, volunteer assistance, and staff friendliness in Taiwan were related to a lower possibility of rating Indonesia’s healthcare as better.

### Overall satisfaction and dimensions of satisfaction

Migrant workers rated high satisfaction with Taiwan’s health care in general and in specific dimensions, and scores were close in all items (with means ranging 4.00–4.39). Age was the only significant factor related to overall satisfaction, and being older was also related to higher satisfaction in the tangible and responsiveness dimensions. This finding aligns with previous studies [23, 36, 37]. It is possible that older migrants are more experienced and have better strategies to handle challenges when accessing healthcare, or older migrants may be more appreciative of good aspects of the healthcare system as socioemotional selectivity theory suggests [38].

Being older, often getting assistance from volunteers, and experiencing higher staff friendliness were related to higher satisfaction with the tangible dimension. Higher satisfaction levels in the tangible dimension suggested that migrant workers may place greater value on the physical aspects of health care, such as facility cleanliness, equipment quality, and overall appearance. Because there is information asymmetry of health care between users and providers, the tangible dimension is easier to evaluate for patients. Migrant workers who received more assistance from volunteers and greater staff friendliness experienced higher satisfaction in the tangible dimension. Assistance from volunteers in hospitals provided more assistance in orientation, which was more likely to increase satisfaction of the patient with the environment and service delivery [39, 40]. In Taiwan, volunteer services are available in many medical centers to help immigrants. This approach can increase patient’s satisfaction, especially for migrant workers who are in need without treating them differently due to of their race, religion, or culture [41]. When migrants often perceive healthcare staff as being friendly, they are more likely to be satisfied with the physical aspects of the healthcare environment, which was also found in previous studies [7, 24]. Staff friendliness is a software aspect of the environment in healthcare settings, that is also easy to evaluate by patients.

Migrant workers who experienced longer waiting time reported lower satisfaction with the empathy of healthcare providers. This result was similar to findings of a previous study [21]. Long waiting time were also identified as the reason for dissatisfaction among migrant population in various countries [5, 42]. Extended waiting periods not only affect patient satisfaction but also contribute to negative emotional responses, including frustration and aggression, which may be directed toward healthcare staff [43]. In Taiwan, waiting time is usually short in clinics, but to make an appointment in regional hospitals or even medical centers, one may need to wait days due to a large number of outpatients. This underscores the need for healthcare systems to prioritize reducing waiting time to enhance patient satisfaction. A pre-registered system and an online application that shows the visiting order with possible waiting time would be helpful in reducing the waiting time.

In the reliable and responsiveness dimensions, only demographic factors were significant. Satisfaction with reliability was related to having no spouse. Having no spouse being related to higher satisfaction was consistent with previous research [32]. No spouse indicates a greater need to rely on healthcare services when getting sick, and thus reliability was especially important for participants without a spouse. Satisfaction with responsiveness was influenced by being female and older, and having a higher income level. Female migrant workers reporting lower satisfaction with responsiveness was also found in a previous study [24]. Females may more fully emphasize comfort, privacy, and safety of the healthcare environment, and thus they are more sensitive to the hospital environment [44]. The context is different in Taiwan, and thus women workers may feel lower responsiveness. It is also possible that women migrant workers have more responsibility in their family, and thus utilizing health care might not be convenient for them when they are in Taiwan due to a smaller family support network. A gender-sensitive, culture-friendly training of professionals and staff may be helpful to Indonesian migrant workers. Higher income being associated with lower satisfaction of the responsiveness dimension is consistent with previous research [45]. Higher-income migrant workers have elevated expectations regarding the quality and timeliness of care, leading to disappointment when such expectations are not met.

### Factors related to comparisons between Taiwan and Indonesia

Comparisons of experiences in the use of healthcare between Taiwan and Indonesia were defined as gaps between perceptions and expectations [3]. In our findings, longer wait time to make an appointment were associated with higher satisfaction with Indonesia’s healthcare tangibility and responsiveness. The confidence intervals of the odds ratios were wide, probably because some cells had smaller sample size (please see the bi-variate analysis in the Appendix Table S2). Thus, the estimation of the parameters may not be stable. The results need to be explained more conservatively. Migrant workers may have had lower expectations for wait times in Indonesia or may have prioritized other aspects of healthcare quality when assessing the overall environment. The results show that more-expensive health service payments were related to less satisfaction with Taiwan’s healthcare tangibility and ways of reliability. Compared with the average expense of outpatient care in Indonesia [46] and Taiwan [47], the out-of-pocket cost can be more expensive in Taiwan than in Indonesia. This indicates that the financial burden of healthcare in Taiwan may detract from an otherwise favorable view of its tangible environment, as found in previous research [48].

It is interesting that when migrant workers needed a translator, they were less satisfied with Taiwan’s healthcare tangibility; but when providers in Taiwan had better language abilities to communicate with them, they were more satisfied with Indonesia’s healthcare tangibility and reliability. A previous study showed that providing access to professional interpreters can significantly improve communication between healthcare providers and patients, leading to better health outcomes and satisfaction [49]. This suggests that effective communication in the healthcare setting, particularly in a familiar language, is critical for improving migrant workers’ perceptions of the healthcare environment. Language barriers in a foreign country can create feelings of isolation or misunderstanding in healthcare settings, and interfere with effective communication between healthcare providers and migrant patient [50]. While patients may appreciate Taiwan’s efficient language services, they might still feel a stronger connection to the cultural familiarity and comfort of Indonesia’s health care, especially if they are familiar with the expected quality of care and conditions. Although healthcare providers and patients speaking the same language or good English can improve patient perceptions of the care quality they receive [51], the providers may not be familiar with the Indonesian culture well. Thus, communication is still ineffective even when speaking the same language. When they experience clear communication in Indonesian, they may feel more comfortable and connected, making them view the environment more positively. Besides language support or translation embedded in the hospitals, introducing culture for migrant workers to staff would be helpful. Getting easy explanations from providers in Taiwan was related to being less satisfied with Indonesia’s trustworthiness. This result was similar to results of another study, although attitudes and communication of health workers were good; however, the satisfaction level of the patient was low [52]. This could be explained by even though workers might receive frequent explanations, the content or quality of the interactions might not meet their expectations. It also suggests that medical professionals should be trained in how to explain the conditions, treatments, and prognosis to migrants in easy English.

Participants who frequently received assistance from volunteers and higher staff friendliness were less likely to rate Indonesia’s healthcare tangibility and reliability as better. Migrant workers may need more assistance with orientation in hospitals, and volunteers may help in the tangible dimension. Previous research emphasized that immigrant patients receiving assistance produced a direct positive effect on healthcare utilization [53]. In Indonesia, the healthcare system is more formalized, and volunteer-driven healthcare support might not be as common compared to countries with more-developed volunteer networks such as Taiwan. A higher staff friendliness and the assistance of volunteers may help the migrant workers feel more comfortable using healthcare in a foreign country. Participants who were crewmen (e.g., fishermen) and those who worked in human social services (mostly domestic workers or in-home care workers) were more likely to rate Indonesia’s tangibility and empathy as better. This result is consistent with another study which found that working as a crewman increased satisfaction with health services [54]. The notable difference could be attributed to their working conditions. Migrant workers in the manufacturing industry may be exposed to health risks related to their occupation. However, they may have regular days off to access healthcare in Taiwan. In contrast, fishermen stay in the ocean for a long time; they may not use healthcare in Taiwan immediately when sick. Domestic or in-home care workers may need to work at night or on weekends. Both groups may face demanding and irregular work schedules in Taiwan, and many hospitals in Taiwan do not provide services on weekends, which could make healthcare appointments or services not be easily accessible during flexible hours. Thus, migrant workers with such healthcare experiences may perceive that Indonesian healthcare systems provide more personal attention.

Higher-income individuals were more likely to rate Taiwan’s healthcare as providing better empathy. This aligned with a previous study which showed that a better income level impacts decision-making, leading to obtaining better hospital utilization [55]. Higher-income workers may have greater access to healthcare services and perceive Taiwan’s healthcare system as being more attentive to their needs. In addition, migrant workers with a higher education were less satisfied with most aspects of Indonesia’s health care and also less satisfied with Taiwan’s health care, consistent with previous research [56]. This dissatisfaction might arise from higher expectations for health care.

## Limitations

This study has some limitations. First, it was a cross-sectional design, meaning that causal relationships could not be confirmed. Second, the measurement of satisfaction with healthcare services was based on retrospective self-reports, which may have introduced recall bias. Third, some confounding factors were not included, such as expectations of care for migrant workers in a foreign country, because the expectation may affect healthcare satisfaction. The study also did not include perspectives of healthcare providers, which could offer valuable insights into factors influencing patient satisfaction and the quality of care delivered. Fourth, there may be challenges in comparing healthcare in two countries for migrant workers. Some migrant workers may not have any experience in using healthcare in Taiwan and Indonesia. Except for the asymmetry of healthcare knowledge, language, and cultural barriers may cause migrant workers to be unable to compare all the dimensions of healthcare quality in a foreign country. The characteristics of certain occupations may prevent migrant workers from using health care regularly, so they may not have much experience with the health care system in Taiwan to rate the quality of care. Fifth, the sample was obtained through purposive sampling, and only certain occupations of migrant workers were considered. The sample cannot be generalized to all kinds of occupations among migrant workers in Taiwan or other societies. A sample with comprehensive occupations of Indonesian migrant workers in different cities is suggested in future research.

## Conclusions

This study highlights significant factors influencing satisfaction with several dimensions of healthcare services among Indonesian migrant workers in Taiwan. Gender, age, marital status, income level, waiting time, and volunteer assistance emerged as significant factors affecting the level of satisfaction with most dimensions of the SERVQUAL scale. When comparing healthcare experiences between Taiwan and Indonesia, waiting time, higher payments, and provider’s language abilities were factors leading to lower satisfaction with Taiwan or higher satisfaction with Indonesia, while easy explanations, assistance from volunteers, and staff friendliness were factors leading to higher satisfaction with Taiwan. These results imply the importance of assistance in the healthcare system for migrant workers to improve their satisfaction, including enhancing cultural competence, implementing more-efficient scheduling systems and queue management to reduce waiting time, and providing friendly attitudes and assistance for migrant workers by hospitals. We suggest volunteer programs and gender-sensitive services (such as female professionals for women patients) be expanded to address the specific needs of migrant workers, providing special night clinics and introducing a pre-registering system for migrant workers with language assistance to reduce waiting time. The healthcare policy should emphasize the training of medical professionals and hospital staff in ethnic/cultural equality and the backgrounds of migrants in providing healthcare.

## Supplementary Information

Supplementary Material 1.

## Data Availability

No datasets were generated or analysed during the current study.
