# Supplementary material for: Satisfaction with healthcare services and related factors among Indonesian migrant workers in Taiwan: a cross-sectional survey study
Source: BMC Health Serv Res. 2025 Apr 23;25:582. doi: 10.1186/s12913-025-12722-9 (PMC12016079; doi:10.1186/s12913-025-12722-9)
Supplement: Supplementary file 1 — Supplementary Material 1. [file 12913_2025_12722_MOESM1_ESM.docx]

Appendix

Table S1. Internal consistency of the SERVQUAL scale

| **Items** | **Cronbach’s Alpha** |
| --- | --- |
| Total | 0.91 |
| **Tangibility** | 0.74 |
| The equipment and facilities are up-to-date |  |
| The physical environment has good signs, symbols and artifacts |  |
| The employees are well-dressed and neat |  |
| The consumable elements used in cure and serve to patients are appealing |  |
| **Reliability** | 0.81 |
| The employees and the health care workers are willing to solve your problems |  |
| The employees and the health care workers do everything right at the first time |  |
| The employees and the health care workers fulfill their promised service at promised time |  |
| The employees and the health care workers keep accurate records and documents |  |
| **Responsiveness** | 0.86 |
| The employees and the health care workers announce the exact time for providing services |  |
| You receive fast and promptly services |  |
| The employees and the heath care workers are willing to help |  |
| The employees are ready to respond to patients’/family members’ enquiries |  |
| **Assurance** | 0.87 |
| The employee and the health care workers are trustworthy |  |
| The employees and the health care workers make patients feel safe |  |
| The employees and the health care workers are polite and humble |  |
| The employees and the health care workers have knowledgeable enough to answer your questions |  |
| **Empathy** | 0.80 |
| The employees and the heath care workers have shown individual attention to patients. |  |
| The hour in health care providers (hospitals or clinics) center is convenient. |  |
| The employees and the heath care workers understand the patients’ specific needs. |  |
| The employees provide services that you need. |  |
| The employees and the health care workers pay attention to all patients equally irrespective of their social status. |  |

Table S2. Bivariate analysis of individual and health care system factors with the comparison of satisfaction of responsiveness, assurance and empathy (Mean (SD) or %)

| Variables | Responsiveness | | | Sig. | Assurance | | | Sig. | Empathy | | | Sig. |
| --- | --- | --- | --- | --- | --- | --- | --- | --- | --- | --- | --- | --- |
|  | Taiwan is better | Indonesia is better | Similar/ it does not apply |  | Taiwan is better | Indonesia is better | Similar/ it does not apply |  | Taiwan is better | Indonesia is better | Similar/ it does not apply |  |
| Individual factors |  |  |  |  |  |  |  |  |  |  |  |  |
| Gender |  |  |  |  |  |  |  |  |  |  |  | * |
| Male | 59.1% | 11.4% | 29.5% |  | 59.8% | 7.6% | 32.6% |  | 62.1% | 12.1% | 25.8% |  |
| Female | 80.7% | 2.8% | 16.5% |  | 68.8% | 1.8% | 29.4% |  | 74.3% | 3.7% | 22.0% |  |
| Age |  |  |  |  |  |  |  |  |  |  |  |  |
| < 30 years | 61.5% | 9.2% | 29.4% |  | 58.7% | 7.3% | 33.9% |  | 58.7% | 11.9% | 29.4% | *** |
| $\geq$30 years | 74.8% | 6.1% | 19.1% |  | 67.9% | 3.1% | 29.0% |  | 74.8% | 5.3% | 19.8% |  |
| Marital status |  |  |  |  |  |  |  |  |  |  |  |  |
| Having spouse | 68.1% | 6.5% | 25.4% |  | 62.3% | 2.9% | 34.8% |  | 71.0% | 7.2% | 21.7% |  |
| No having spouse | 69.3% | 8.9% | 21.8% |  | 65.3% | 7.9% | 26.7% |  | 62.4% | 9.9% | 27.7% |  |
| Occupation |  |  |  | *** |  |  |  |  |  |  |  |  |
| Manufacturing | 59.6% | 6.4% | 33.9% |  | 61.5% | 4.6% | 33.9% |  | 62.4% | 10.1% | 27.5% |  |
| Crewman | 66.7% | 19.4% | 13.9% |  | 58.3% | 13.9% | 27.8% |  | 61.1% | 16.7% | 22.2% |  |
| Human social service | 80.2% | 4.2% | 15.6% |  | 68.8% | 2.1% | 29.2% |  | 76.0% | 3.1% | 20.8% |  |
| Income level |  |  |  |  |  |  |  |  |  |  |  |  |
| < $635 USD /month | 66.7% | 4.8% | 28.6% |  | 52.4% | 2.4% | 45.2% |  | 54.8% | 7.1% | 38.1% |  |
| $\geq$ $635 USD /month | 69.7% | 8.1% | 22.2% |  | 66.7% | 5.6% | 27.8% |  | 70.7% | 8.6% | 20.7% |  |
| Educational Level |  |  |  | * |  |  |  |  |  |  |  | * |
| Junior high school or lower | 67.4% | 13.0% | 19.6% |  | 68.5% | 7.6% | 23.9% |  | 73.9% | 10.9% | 15.2% |  |
| Senior high school or higher | 70.2% | 4.3% | 25.5% |  | 61.0% | 3.5% | 35.5% |  | 63.1% | 7.1% | 29.8% |  |
| Taiwan NHI status |  |  |  |  |  |  |  |  |  |  |  |  |
| Yes | 68.7% | 7.3% | 24.0% |  | 64.4% | 5.2% | 30.5% |  | 68.2% | 8.2% | 23.6% |  |
| No/I don’t know | 75.0% | 12.5% | 12.5% |  | 50.0% | 0% | 50.0% |  | 50.0% | 12.5% | 37.5% |  |
| Time spent in Taiwan |  |  |  |  |  |  |  |  |  |  |  |  |
| ≤ 2 years | 66.7% | 8.7% | 24.6% |  | 58.0% | 7.2% | 34.8% |  | 59.4% | 8.7% | 31.9% |  |
| > 2 years | 70.9% | 7.3% | 21.8% |  | 66.7% | 4.2% | 29.1% |  | 70.9% | 8.5% | 20.6% |  |
| Translator needed |  |  |  | ** |  |  |  |  |  |  |  |  |
| No | 79.0% | 6.7% | 14.3% |  | 68.9% | 3.4% | 27.7% |  | 72.3% | 6.7% | 21.0% |  |
| Yes | 59.0% | 8.2% | 32.8% |  | 59.0% | 6.6% | 34.4% |  | 63.1% | 9.8% | 27.0% |  |
| Physical health |  |  |  |  |  |  |  |  |  |  |  |  |
| Poor | 61.0% | 11.9% | 27.1% |  | 54.2% | 8.5% | 37.3% |  | 57.6% | 10.2% | 32.2% |  |
| Good | 71.3% | 6.1% | 22.7% |  | 66.9% | 3.9% | 29.3% |  | 70.7% | 7.7% | 21.5% |  |
| Mental health |  |  |  |  |  |  |  |  |  |  |  |  |
| Poor | 70.0% | 7.5% | 22.5% |  | 62.5% | 5.8% | 31.7% |  | 66.7% | 10.0% | 23.3% |  |
| Good | 67.5% | 7.5% | 25.0% |  | 65.0% | 4.2% | 30.8% |  | 68.3% | 6.7% | 25.0% |  |
| Healthcare system factors |  |  |  |  |  |  |  |  |  |  |  |  |
| Waiting time to make an appointment |  |  |  |  |  |  |  |  |  |  |  |  |
| Less than one day | 68.9% | 6.7% | 24.4% |  | 63.6% | 4.9% | 31.6% |  | 68.9% | 7.6% | 23.6% |  |
| More than one day | 68.8% | 18.8% | 12.5% |  | 68.8% | 6.3% | 25.0% |  | 50.0% | 18.8% | 31.3% |  |
| Counselling time |  |  |  |  |  |  |  |  |  |  |  |  |
| Less than one hour | 68.2% | 7.4% | 24.4% |  | 63.1% | 5.1% | 31.8% |  | 67.7% | 8.8% | 23.5% |  |
| More than one hour | 75.0% | 8.3% | 16.7% |  | 70.8% | 4.2% | 25.0% |  | 66.7% | 4.2% | 29.2% |  |
| Need to wait the doctor (long) | 2.15 (0.65) | 2.05 (0.51) | 2.11 (0.52) |  | 2.15 (0.63) | 2.28 (0.83) | 2.07 (0.49) |  | 2.15 (0.63) | 2.26 (0.81) | 2.06 (0.49) |  |
| Time of transportation (long) | 2.17 (0.73) | 2.10 (0.79) | 2.05 (0.44) |  | 2.15 (0.69) | 2.28 (0.96) | 2.07 (0.55) |  | 2.18 (0.71) | 2.26 (0.93) | 2.00 (0.46) |  |
| Health service payment (expensive) | 2.01 (0.58) | 2.30 (1.08) | 2.09 (0.51) |  | 2.00 (0.54) | 2.44 (1.20) | 2.06 (0.56) | * | 2.02 (0.59) | 2.37 (1.21) | 2.03 (0.42) |  |
| Easy explanations (often) | 3.62 (1.26) | 3.50 (1.24) | 3.34 (1.04) |  | 3.64 (1.23) | 3.11 (1.28) | 3.45 (1.12) |  | 3.67 (1.22) | 3.26 (1.28) | 3.34 (1.14) |  |
| Language (good) | 3.70 (0.73) | 3.55 (0.76) | 3.55 (0.60) |  | 3.71 (0.71) | 3.56 (0.98) | 3.55 (0.60) |  | 3.71 (0.71) | 3.53 (0.77) | 3.56 (0.66) |  |
| Translator provided (often) | 2.17 (1.30) | 2.55 (1.67) | 2.07 (1.25) |  | 2.18 (1.30) | 2.22 (1.44) | 2.15 (1.35) |  | 2.16 (1.30) | 2.42 (1.30) | 2.13 (1.39) |  |
| Assist by volunteers (often) | 2.39 (1.29) | 2.35 (1.18) | 2.59 (1.35) |  | 2.36 (1.27) | 2.22 (1.00) | 2.63 (1.39) |  | 2.35 (1.30) | 2.63 (1.21) | 2.56 (1.30) |  |
| Friendliness of staff (good) | 4.06 (0.57) | 3.95 (0.51) | 4.03 (0.37) |  | 4.06 (058) | 4.00 (059) | 4.03 (033) |  | 4.04 (0.60) | 3.89 (0.46) | 4.10 (0.31) |  |

Note: N=241. Analysis bivariate analysis was conducted by Chi-square or one-way ANOVA, *p<0.05, **p<0.01, ***p<0.001.
